# Consensus Pharmacological Interactions for PLK2 Inhibitor Identification in Colorectal Cancer Treatment

**DOI:** 10.1021/acs.jcim.5c02197

**Published:** 2025-12-17

**Authors:** Yi-Wen Wu, Chun-Lin Yang, Tony Eight Lin, Yun-Hsuan Yeh, Yu-Ting Fang-Chin, Tzu-Ying Sung, Shih-Chung Yen, Jui-Hua Hsieh, Cheng-Chih Chung, Shiow-Lin Pan, Kai-Cheng Hsu

**Affiliations:** † Graduate Institute of Cancer Biology and Drug Discovery, College of Medical Science and Technology, 38032 Taipei Medical University, Taipei 11031, Taiwan; ‡ Ph.D. Program for Cancer Molecular Biology and Drug Discovery, College of Medical Science and Technology, Taipei Medical University, Taipei 11031, Taiwan; § Warshel Institute for Computational Biology, School of Medicine, 407605 The Chinese University of Hong Kong (Shenzhen), Shenzhen, Guangdong 518172, China; ∥ Division of Translational Toxicology, National Institute of Environmental Health Sciences, 551251 National Institutes of Health, Durham, North Carolina 27709, United States; ⊥ Division of Cardiology, Department of Internal Medicine, School of Medicine, College of Medicine, Taipei Medical University, Taipei 11031, Taiwan; # Division of Cardiovascular Medicine, Department of Internal Medicine, Wan Fang Hospital, Taipei Medical University, Taipei 11696, Taiwan; ∇ TMU Research Center of Cancer Translational Medicine, Taipei Medical University, Taipei 11031, Taiwan; ○ Cancer Center, Wan Fang Hospital, Taipei Medical University, Taipei 11031, Taiwan

## Abstract

PLK2 plays a critical role in cellular stress response, redox regulation, and tumor progression. In colorectal cancer (CRC), elevated PLK2 expression is associated with chemoresistance and poor patient prognosis, making it a compelling target for therapeutic intervention. In this study, we used a structure-based drug discovery strategy to develop a consensus model incorporating pharmacological interactions from various PLK2 structures. This model enhanced the hit rate for identifying inhibitors during virtual screening, increasing the ROC-AUC from 0.906 to 0.930. We then used the model to screen the ChemDiv compound library and identified two novel PLK2 inhibitors. Next, we searched for analogs of the most potent compound and evaluated their activity. Two analogs demonstrated submicromolar inhibition, including Y207–5465 (IC_50_: 584.3 nM) and 8012–3246 (IC_50_: 774.5 nM). Structure–activity relationship (SAR) analysis was performed to identify key interactions contributing to potency. In vitro assays demonstrated that 8012–3246 exhibited better cytotoxicity (IC_50_: 7.97 and 17.67 μM) and antiproliferative effects (GI_50_: 3.28 and 6.62 μM) in HT-29 and HCT-116 CRC cell lines, respectively. Kinase profiling confirmed that 8012–3246 possesses high selectivity for PLK2. Mechanistic studies further revealed that 8012–3246 inhibited GSK3β phosphorylation, a key downstream effector of PLK2 involved in redox homeostasis and cell survival. These findings support the use of pharmacological consensus modeling to identify novel PLK2 inhibitors and highlight PLK2 inhibition as a promising strategy for CRC treatment.

## Graphical abstract

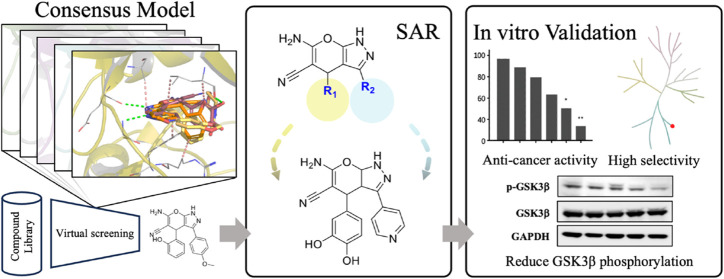

## Introduction

Polo-like kinase 2 (PLK2), a member of the Polo-like kinase family (PLK1-PLK5), is a serine/threonine kinase that plays a crucial role in cell cycle regulation and cellular stress response, particularly outside of mitosis.^1^ PLK2 has emerged as a critical regulator connecting cell cycle control with oncogenic signaling.^1,2^ Predominantly active during the G1 and S phases, PLK2 participates in initiating centrosome duplication and facilitating early cell cycle progression.^3,4^ Recent studies have shown that PLK2 promotes tumor progression by activating mutant p53, enhancing chemoresistance, and driving cancer cell proliferation, while its downregulation in certain contexts suggests a potential tumor-suppressive role.^5,6^ These findings highlight PLK2 as a key regulator of genomic maintenance and stress signaling, suggesting its potential involvement in tumorigenesis and its promise as a therapeutic target in cancer treatment.

Recent studies have further implicated PLK2 as a context-dependent oncogene in various solid tumors, including colorectal, pancreatic, and ovarian cancers.^7,8^ While initially considered a tumor suppressor via its engagement in the p53-mediated DNA damage response, PLK2 has been shown to promote tumor progression in settings characterized by oxidative stress or dysfunctional p53 signaling.^9^ In cancer cells, PLK2 supports survival by enhancing chemoresistance and redox adaptation through phosphorylation of key downstream effectors such as GSK3β and NRF2. Specifically, PLK2 phosphorylates GSK3β at Ser9, suppressing its activity and facilitating NRF2 nuclear translocation.^10^ Additionally, PLK2 phosphorylates NRF2 at Ser40, strengthening its interaction with p21^Cip1^ and activating cytoprotective gene expression. These modifications enhance mitochondrial stability and attenuate p53-dependent necrosis, allowing cancer cells to persist under metabolic and oxidative stress.^11^ In colorectal cancer (CRC), the PLK2/GSK3β/NRF2 signaling axis plays a critical role in regulating redox homeostasis and promoting chemoresistance.^12^ By enhancing NRF2 activation, PLK2 supports the expression of antioxidant genes, allowing cancer cells to survive under oxidative and therapeutic stress.^11^ Clinically, elevated PLK2 expression has been linked to poorer treatment response and reduced overall survival in CRC patients.^6^ These findings highlight PLK2 as a promising molecular target for therapeutic intervention in CRC and other PLK2-driven malignancies.

Several PLK2 inhibitors have been developed in recent years.^13,14^ In cancer therapy, the pan-PLK inhibitor BI-2536 has completed phase II evaluation and has demonstrated antiproliferative activity across various solid tumors.^15^ In addition, compounds C2 and C21 suppress cancer cell growth,^16^ and compound 7AO (ON1231320) induces mitotic arrest and apoptosis, highlighting their therapeutic potential.^4,17^ However, most of these compounds exhibit limited selectivity. For instance, BI-2536 inhibits CAMKIIa, SYK, and STK16, leading to potential off-target effects.^16^ Therefore, designing new PLK2 inhibitors with high selectivity is critical for future therapeutic applications.

In this study, we aimed to identify novel PLK2 inhibitors using structure-based virtual screening, followed by experimental validation through enzyme- and cell-based assays (Figure 1). First, all available PLK2 crystal structures were evaluated by redocking their cocrystallized ligands. Consensus interactions shared by multiple inhibitors were defined as pharmacological interactions. Previous studies have shown that identifying interactions essential for ligand-kinase binding helps improve the hit rate of virtual screening.^18–20^ Therefore, we identified key pharmacological interactions for each structure using known PLK2 inhibitors. In addition, we further established a consensus model that integrates pharmacological interactions from multiple structures to improve screening accuracy. Using this model, the ChemDiv compound library was screened, and top-ranking candidates were selected and tested for PLK2 inhibition using enzymatic assays. The most active compound was further used to identify analogs for structure–activity relationship (SAR) analysis and selectivity profiling. Finally, validated inhibitors were assessed for their effects on colon cancer cell viability and proliferation. In summary, this study identified novel PLK2 inhibitors that serve as promising starting points for the development of PLK2-targeted cancer therapeutics.

**Figure 1:**
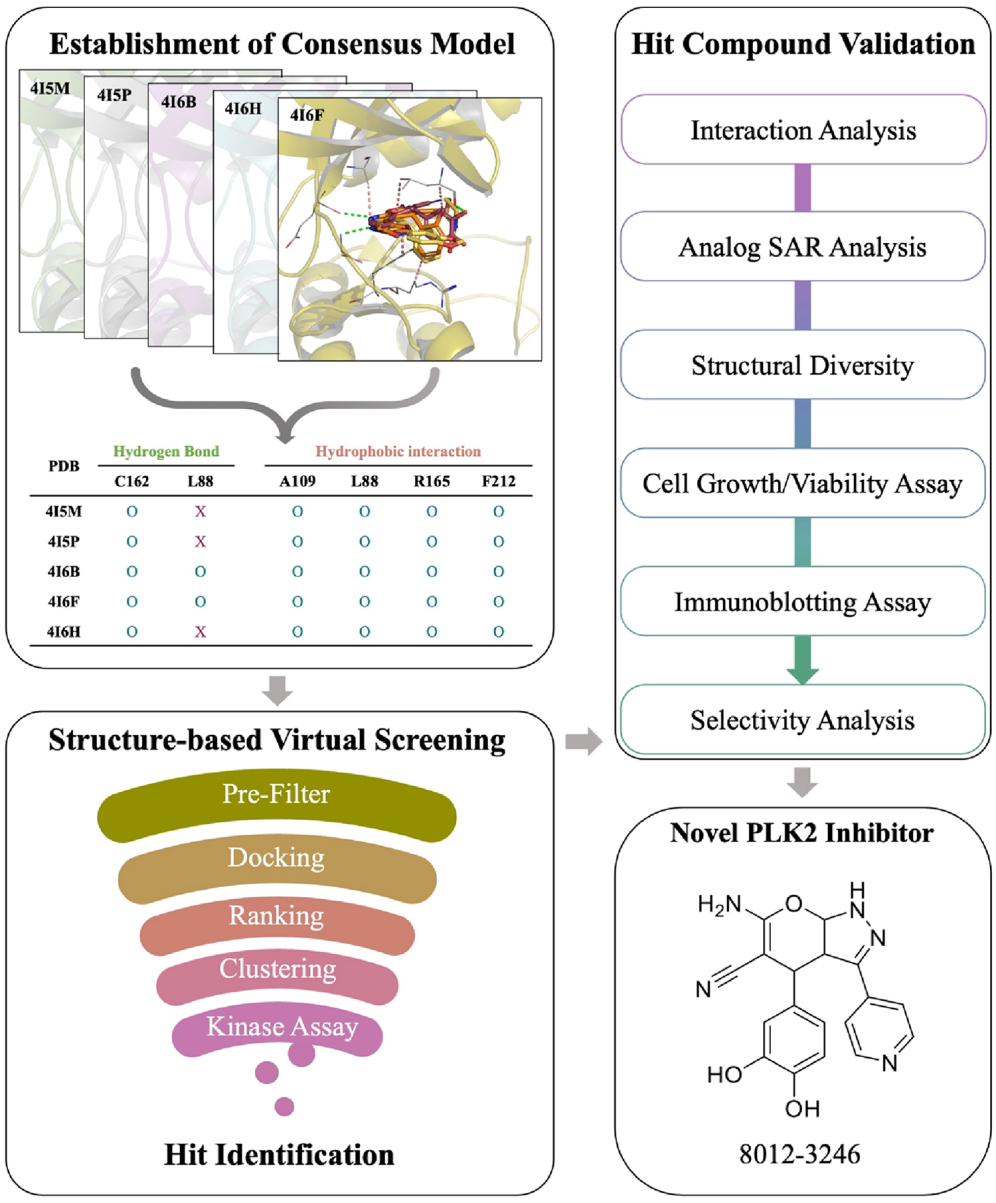
Workflow of the study. A computational screening model for identifying PLK2 inhibitors was first established. Then, the model was applied to identify potential inhibitors. Selected compounds were validated using a kinase assay. The effects of identified inhibitors on CRC were further evaluated via cell-based assays.

## Materials and Methods

### Molecular Docking

Molecular docking was performed using Maestro with default settings.^21^ Crystal structures of PLK2 were obtained from the RCSB Protein Data Bank (PDB).^22^ The structures were prepared using the Protein Preparation Wizard by adding hydrogen atoms, assigning partial charges to residues, and removing water molecules. A docking grid for each structure was generated using the Receptor Grid Generation module, centered on the cocrystallized ligand. Compounds were prepared for docking using the LigPrep module by generating 3D structures and assigning hydrogen atoms and charges. Docking was performed using GLIDE.^23^ Interactions between compounds and protein residues were analyzed using Pipeline Pilot.^24^

### Establishment of Consensus Model

Known PLK2 inhibitors with half-maximal inhibitory concentration (IC_50_) values below 10 μM were collected from the ChEMBL database^25^ to identify key pharmacological interactions. From this set, 60 structurally diverse compounds were selected for docking analysis. These compounds were docked into the binding sites of each selected PLK2 structure. Based on docking scores, the top 30 inhibitors for each structure were used to identify key pharmacological interactions. Interaction analysis was performed using Pipeline Pilot. Pharmacological interactions were defined as hydrogen-bonding interactions observed in at least 50% of the docked inhibitors and hydrophobic interactions observed in at least 80% of the docked inhibitors. A pharmacological score was calculated for each compound using the following equation:

S(i)=N(i)+(−0.01)×D(i)

where *S*(*i*) is the pharmacological score of compound *i*, *N*(*i*) is the number of pharmacological interactions formed by compound *i*, and *D*(*i*) is the docking score of compound *i*. Compounds were ranked based on their pharmacological scores. To generate a consensus model, rankings from individual PLK2 structures were integrated. For each compound, rankings across two or more structures were summed to calculate a consensus score. Compounds were then reranked based on this consensus score, and the final ranking was used to prioritize potential PLK2 inhibitors.

### Preparation of Screening Library

Virtual screening was performed using the ChemDiv compound library, which contains approximately 1.6 million compounds. The compounds were first preprocessed using Pipeline Pilot. A high-throughput screening filter was then applied to remove compounds containing nonorganic atoms or reactive substructures. Next, compounds that violated Lipinski’s Rule of Five^26^ or Veber’s criteria,^27^ or contained pan-assay interference compounds (PAINS),^28^ were excluded. In addition, compounds with a quantitative estimate of drug-likeness (QED) score below 0.25 were removed. The remaining compounds were used for the virtual screening of potential PLK2 inhibitors.

### Kinase Assay

Kinase inhibitory activity was measured using a fluorescence resonance energy transfer (FRET)-based LanthaScreen Eu Kinase Binding Assay (Thermo Fisher Scientific). In this assay, a fluorescent acceptor dye is bound to the kinase, and an Eu-labeled antitag antibody is added to detect a phosphorylated, fluorescently labeled substrate. The fluorescently labeled kinase, antibody, and test compound were combined in an appropriate buffer and incubated. After incubation, the mixture was placed into a fluorescence plate reader capable of detecting FRET signals. FRET efficiency was calculated by comparing the ratio of acceptor emission to donor emission. The selected compounds were evaluated at specified concentrations, and IC_50_ values were determined using GraphPad Prism software. Each kinase activity assay was performed in duplicate and followed Thermo Fisher Scientific’s quality control guidelines. Kinase profiling was conducted using Thermo Fisher’s SelectScreen Kinase service, with supplemental assay formats (Adapta and Z′LYTE) employed as needed. Detailed information on these assay protocols is available on the Thermo Fisher Scientific Web site (Adapta: www.thermofisher.com/adapta; Z′LYTE: www.thermofisher.com/z-lyte). The selectivity of the compound was evaluated using the Eurofins kinase profiling service.

### Cell Culture

Human CRC cell line HT-29 was obtained from American Type Culture Collection (HTB-38, RRID: CVCL_0320, ATCC, VA), while HCT-116 was sourced from Bioresource Collection and Research Center (Cat#60349, RRID: CVCL_0291, BCRC, Hsinchu, Taiwan). Both cell lines were cultured in McCoy’s 5A medium supplemented with 10% (v/v) fetal bovine serum (FBS), 100 units/mL of penicillin, and 100 μg/mL of streptomycin. Cells were maintained in a humidified incubator at 37 °C with 5% CO_2_.

### Cell Viability Analysis

Cells were seeded in 96-well plates at a density of 3.5 × 10^3^ cells/well. After allowing the cells to adhere overnight, they were treated with concentrations of test compounds at 0.3, 1, 3, 10, and 30 μM for 72 h. Following treatment, cell viability was assessed using the 3-(4,5-Dimethylthiazol-2-yl)-2,5-diphenyltetrazolium bromide (MTT) assay. MTT reagent (0.5 mg/mL in phosphate-buffered saline (PBS)) was added to each well at a 1:10 volume ratio, and the plates were incubated at 37 °C for 1 h. Subsequently, 100 μL of dimethyl sulfoxide (DMSO) was added to each well to solubilize the formazan crystals formed by viable cells. The absorbance was measured at 550 nm using a microplate reader (Synergy HTX ELISA reader, Bioteck, CA). The IC_50_ values were calculated based on the cell viability.

### BrdU Cell Proliferation Assay

To evaluate the effect of test compounds on cell proliferation, a BrdU Cell Proliferation Assay Kit (MerckMillipore, Darmstadt, Germany) was used. Cells were seeded in 96-well plates at a density of 3.5 × 10^3^ cells/well. After allowing the cells to adhere overnight, they were treated with concentrations of test compounds at 0.3, 1, 3, 10, and 30 μM for 72 h. Twenty-4 h before the end point, 10 μM of BrdU was added to each well and incubated at 37 °C to label newly synthesized DNA. After incubation, the BrdU-containing medium was removed, and cells were fixed with 200 μL of fixing solution for 30 min at room temperature. Wells were washed three times with wash buffer, followed by the addition of 100 μL of anti-BrdU monoclonal antibody. After washing, 100 μL of goat antimouse IgG secondary antibody was added to each well. Following additional washes, 100 μL of TMB substrate was added to develop color. Absorbance was measured at 450 nm using a microplate reader. Cell proliferation was quantified based on BrdU incorporation, and the half-maximal growth inhibitory concentration (GI_50_) values were calculated accordingly.

### Colony Formation Assay

Cells were seeded in 6-well plates at a density of 1 × 10^3^ cells per well and allowed to adhere overnight. The following day, cells were treated with the indicated concentrations of the test compound and incubated for 12 days to allow colony formation. After treatment, the culture medium was removed, and cells were gently washed with PBS. Colonies were then stained with 0.5% crystal violet solution for 20 min at room temperature. Excess stain was removed by washing under running water, and the plates were left to air-dry. Colonies were then counted either visually by the naked eye under a light microscope, with only clusters containing more than 50 cells considered as valid colonies.

### Western Blotting

Cells were seeded in 6 cm dishes at a density of 5 × 10^5^ cells/well and allowed to adhere overnight. Cells were then treated with the indicated concentrations of the test compound for 2 h. Following treatment, total protein lysates were collected using RIPA buffer supplemented with phosphatase inhibitors (2 mM Na_3_VO_4_, 1 mM NaF, and 20 mM NaP_2_O_4_) and an EDTA-free protease inhibitor cocktail. Lysates were centrifuged at 14,000 rpm for 15 min at 4 °C, and protein concentrations were determined using a BCA protein assay kit.

For sample preparation, protein lysates were mixed with sample buffer (312.5 mM Tris-HCl, pH 6.8, 10% SDS, 50% glycerol, 0.05% bromophenol blue, and 10% 2-mercaptoethanol) and denatured at 95 °C for 10 min. Equal amounts of protein were resolved by SDS-PAGE and transferred onto PVDF membranes. Membranes were blocked with 5% nonfat milk in TBST (Tris-buffered saline with 0.1% Tween-20) for 1 h at room temperature, followed by overnight incubation at 4 °C with primary antibodies diluted in TBST. The next day, membranes were washed with TBST and incubated with horseradish peroxidase (HRP)-conjugated secondary antibodies for 1 h at room temperature. The following primary antibodies were used: phospho-GSK3β (Ser9) and total GSK3β (Cell Signaling Technology, MA), and GAPDH (MAB374, Millipore, Bedford, MA). HRP-conjugated secondary antibodies antirabbit IgG (111-035-003) and antimouse IgG (115-035-003) were obtained from Jackson ImmunoResearch (PA). Protein bands were visualized using an enhanced chemiluminescence (ECL) detection kit and imaged with an eBLOT system. Quantification of protein expression levels was performed using ImageJ software.

### Similarity Matrix

Molecules were processed using RDKit^29^ in Python. The ECFP was used with a radius of 2 and a bit size of 2048. A total of 30 structurally diverse molecules from the ChEMBL library were picked. Adding the hit molecules, a Tanimoto^30^ similarity matrix between each molecule pair was generated. A cluster map of the results was then generated using the Python plotting package Seaborn.^31^

### Statistical Analysis

Quantitative statistical tests were applied only when each experimental group consisted of at least five samples. Results from groups with fewer than five replicates are presented for descriptive purposes and indicated as qualitative data. Data are presented as the mean ± standard deviation (SD) or as a percentage of the control, as appropriate for the data set. All statistical analyses were performed using Prism 10 (Graphpad Inc.). Differences between experimental and control groups were analyzed using Student’s *t* test (two-tailed when applicable), with a threshold of *p* < 0.05 considered statistically significant. For comparisons among more than two groups, one-way analysis of variance (ANOVA) was used. When ANOVA indicated a significant difference, Tukey’s post hoc test was performed to determine which specific group differences were significant (using *p* < 0.05 as the significance criterion).

## Results

### Evaluation of Docking Protocol and PLK2 Structures

To identify PLK2 protein structures suitable for virtual screening, all available PLK2 structures were obtained from the PDB for redocking analysis. Each of these structures contains a cocrystallized ligand within the binding site. This approach was used to evaluate the structural reliability of each structure by assessing its ability to reproduce the cocrystallized binding pose using our docking protocol. The retrieved PLK2 structures included five PDB entries: 4I5M, 4I5P, 4I6B, 4I6F, and 4I6H. The cocrystallized ligands were extracted and redocked into their corresponding binding sites, and the root-mean-square deviation (RMSD) between the redocked and crystal poses was calculated. A redocked pose was considered successful when the RMSD was below 2.5 Å, which is a commonly used threshold for redocking.^32^ All redocked poses achieved RMSD values below 2.5 Å (Table S1), and 4I6F exhibited the lowest RMSD value (0.75 Å). These results confirm the suitability of the structures for subsequent analysis and the reliability of the docking protocol in predicting compound binding poses.

### Identification of Pharmacological Interactions

We next identified pharmacological interactions within the PLK2 binding site by analyzing residues that frequently interact with known inhibitors, aiming to improve hit rates in virtual screening. A total of 60 structurally diverse known PLK2 inhibitors were docked into each structure, and the top 30 inhibitors ranked by docking score were selected to calculate the frequencies of hydrogen-bonding and hydrophobic interactions. Pharmacological hydrogen bonds were defined as those formed with at least 50% of the docked inhibitors (Figure 2A), while pharmacological hydrophobic interactions were defined as those occurring in at least 80% of the molecules (Figure 2B).

**Figure 2:**
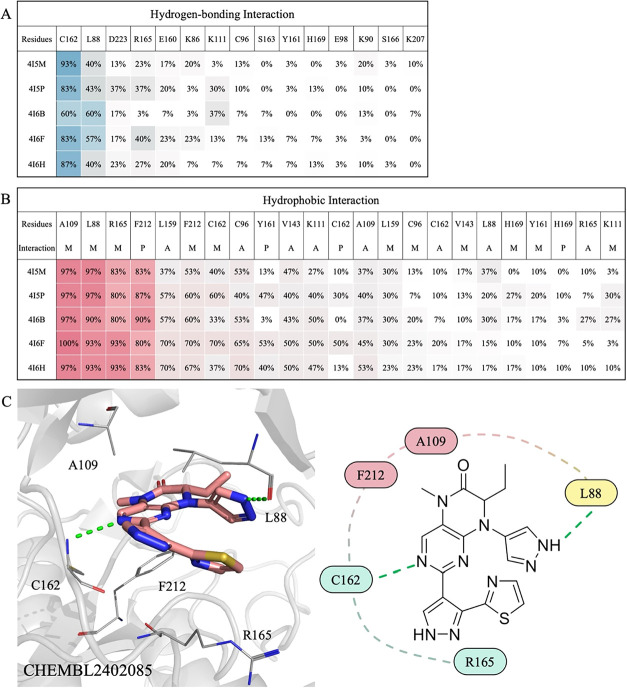
Pharmacological interaction profiles across five PLK2 structures. (A) Frequency of hydrogen-bonding interactions. Residues forming hydrogen bonds with more than 50% of known PLK2 inhibitors are highlighted in blue. (B) Frequency of hydrophobic interactions. Residues engaged in hydrophobic interactions with more than 80% of known inhibitors are shown in red. A, P, and M stand for alkyl hydrophobic interaction, pi-stacking interaction, and mixed hydrophobic interaction, respectively. (C) Docking pose of CHEMBL2402085 within the PLK2 binding pocket, illustrating pharmacological interactions. Hydrogen bonds are depicted as green dashed lines; binding site residues are rendered as labeled sticks. Residues from the hinge, interior, and exterior regions are colored blue, red, and yellow, respectively.

Both common and structure-specific interaction patterns were observed across the PLK2 structures. In most structures, inhibitors frequently formed hydrogen bonds with residue C162, accompanied by consistent hydrophobic interactions involving L88, A109, R165, and F212 (Figure 2B). Structure 4I6F was selected as a representative example to illustrate these interactions. The docking pose of compound CHEMBL2402085 in 4I6F demonstrated these key interactions (Figure 2C). Notably, in both 4I6B and 4I6F, an additional hydrogen bond was frequently formed with residue L88. These findings indicate that most interaction patterns are conserved across the structures; however, slight differences in interaction frequencies were observed. These variations may result from structural differences between the structures, suggesting that incorporating multiple protein structures could be beneficial for virtual screening.

### Establishment of Pharmacological Model

To further evaluate the effectiveness of pharmacological interactions in virtual screening, we established a pharmacological model for each PLK2 structure. A set of 30 known CLK4 inhibitors and 990 randomly selected compounds from the Available Chemical Directory (ACD) were docked into the PLK2 binding sites.^33^ Each compound was evaluated using two scoring approaches: the standard docking score and a pharmacological score, which combined the docking score with the number of key interactions formed by the compound. Model performance in distinguishing true inhibitors from decoys was assessed using the area under the receiver operating characteristic curve (AUC). The results showed that ranking compounds by pharmacological scores consistently yielded higher AUC values than ranking by docking scores alone (Table S1). Among the five PLK2 structures analyzed, 4I5M, 4I5P, and 4I6F demonstrated the best performance, with pharmacological score AUCs exceeding 0.90. Notably, 4I5P achieved the highest AUC value (0.918), indicating its superior ability to distinguish true inhibitors from random compounds. These findings confirm that incorporating pharmacological interactions improves virtual screening performance.

Following the individual model evaluations, we investigated whether combining predictions from multiple pharmacological models could further enhance screening performance. The top three performing models, specifically 4I5M, 4I5P, and 4I6F, were selected for ensemble evaluation. Pairwise consensus models were constructed by summing the individual pharmacological rankings of each compound across models (e.g., 4I5M + 4I5P). For each compound, ranks from these models were added to generate a consensus score, which was then used for reranking. AUC analysis based on these consensus rankings showed improved performance compared to using individual models alone (Table S2). Notably, integrating all three models yielded the highest AUC value of 0.930, representing the best overall performance observed. These findings indicate that a consensus model leveraging complementary pharmacological features can substantially improve virtual screening accuracy. Based on these results, the consensus model was selected as the final framework for subsequent screening.

### Identification of Potential Inhibitors

We applied the consensus model to identify potential PLK2 inhibitors from the ChemDiv database. Compounds were first filtered by removing those containing PAINS structural motifs, a QED score below 0.24, or violations of Lipinski’s rule of five or Veber’s criteria. The remaining compounds were docked into the three selected PLK2 crystal structures. Based on docking scores, the top 5000 compounds were initially selected based on their docking scores. For each of these compounds, pharmacological scores were calculated and ranked across the three protein structures. The ranks were used to generate a consensus score for reranking the compounds. The top 500 consensus-ranked compounds were then clustered based on structural similarity. Fourteen representative compounds were selected from each cluster based on availability for experimental testing.

The selected compounds were evaluated for their inhibitory activity against PLK2 using a kinase assay at a concentration of 3 μM. Among the tested compounds, Y207-5465 exhibited the most potent inhibition, reaching 97% (Table 1). To further explore this scaffold, a series of structurally related analogs was assessed. Notably, compound 8012-3246 also demonstrated strong activity, with 74% inhibition (Table 2). In contrast, the remaining analogs displayed substantially lower activity, with inhibition rates below 25%. To further validate their potency, IC_50_ values were determined for Y207-5465 and 8012-3246. Y207-5465 exhibited an IC_50_ value of 584.3 nM, while 8012-3246 showed an IC_50_ of 774.5 nM (Table 3 and Figure S1). Collectively, these results suggest that our screening strategy is effective for identifying novel PLK2 inhibitors. In particular, Y207-5465 and 8012-3246 represent promising inhibitors for further development.

**Table 1:**
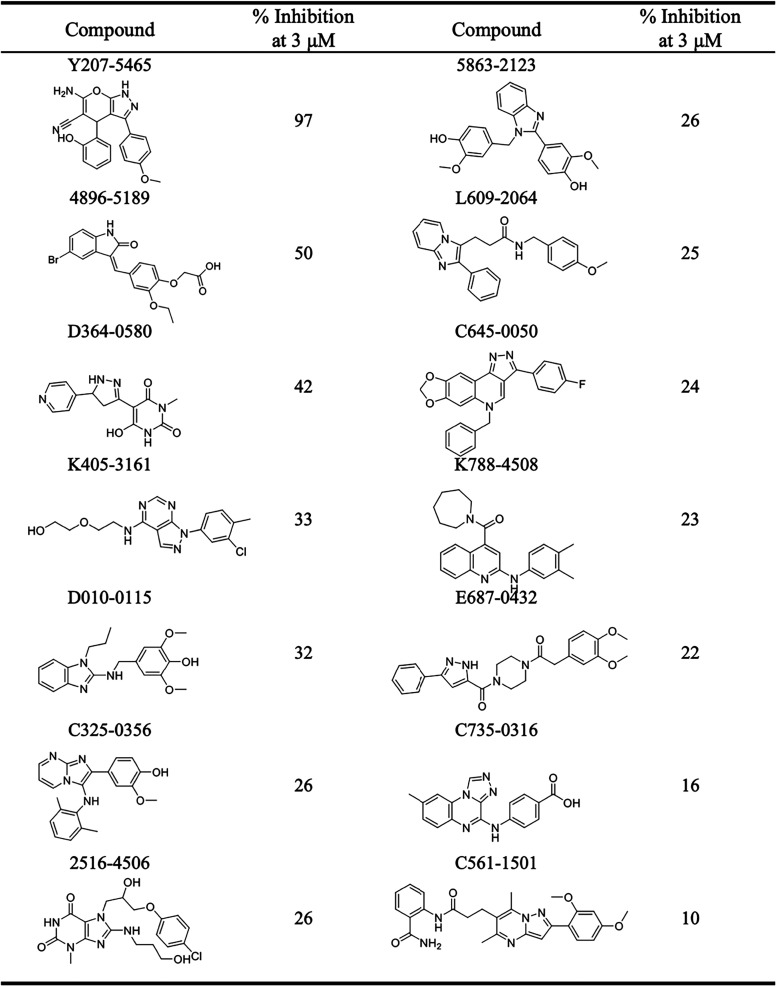
Inhibitory Activity of Selected Compounds

**Table 2:**
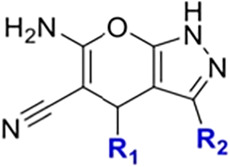

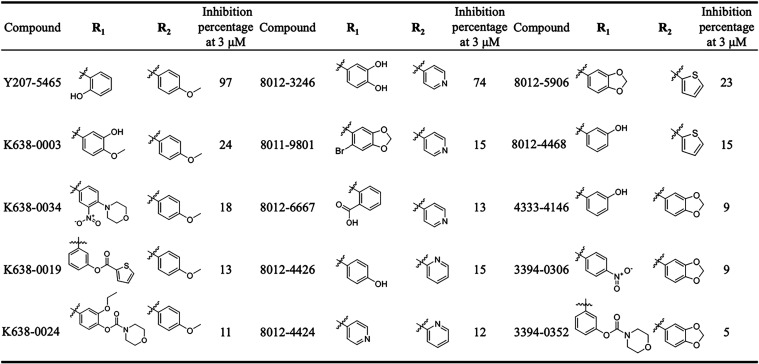
Inhibitory Activity of Y207-5465 Analogs

**Table 3:** IC_50_ Values of Y207-5465 and 8012-3246

**compound name**	**IC**_**50**_ **(nM)**
Y207-5465	584.3
8012-3246	774.5

### Interactions of Identified Inhibitors

We conducted an interaction analysis to investigate the molecular interactions of compounds Y207-5465 and 8012-3246 (Figure 3). Each compound can be divided into three structural regions: the core, the R_1_ substituent, and the R_2_ substituent. Both compounds share a common pyrazolopyrimidine core, which anchors the molecule in the ATP-binding site by forming two hydrogen bonds with hinge residues E160 and C162. The core is further stabilized by hydrophobic interactions with residues A109 and F212. The R_1_ substituents of both compounds are structurally similar and establish a hydrogen bond and hydrophobic contacts with residue L88. The primary structural difference lies in the R_2_ group. In Y207-5465, the 4-methoxyphenyl moiety forms hydrophobic interactions with residues Y161, C162, and R165. Similarly, 8012-3246 contains a pyridyl group at R_2_, which retains interactions with C162 and R165 but lacks contact with Y161. This reduced number of interactions may contribute to the slightly higher IC_50_ value of 8012-3246 compared to Y207-5465.

**Figure 3:**
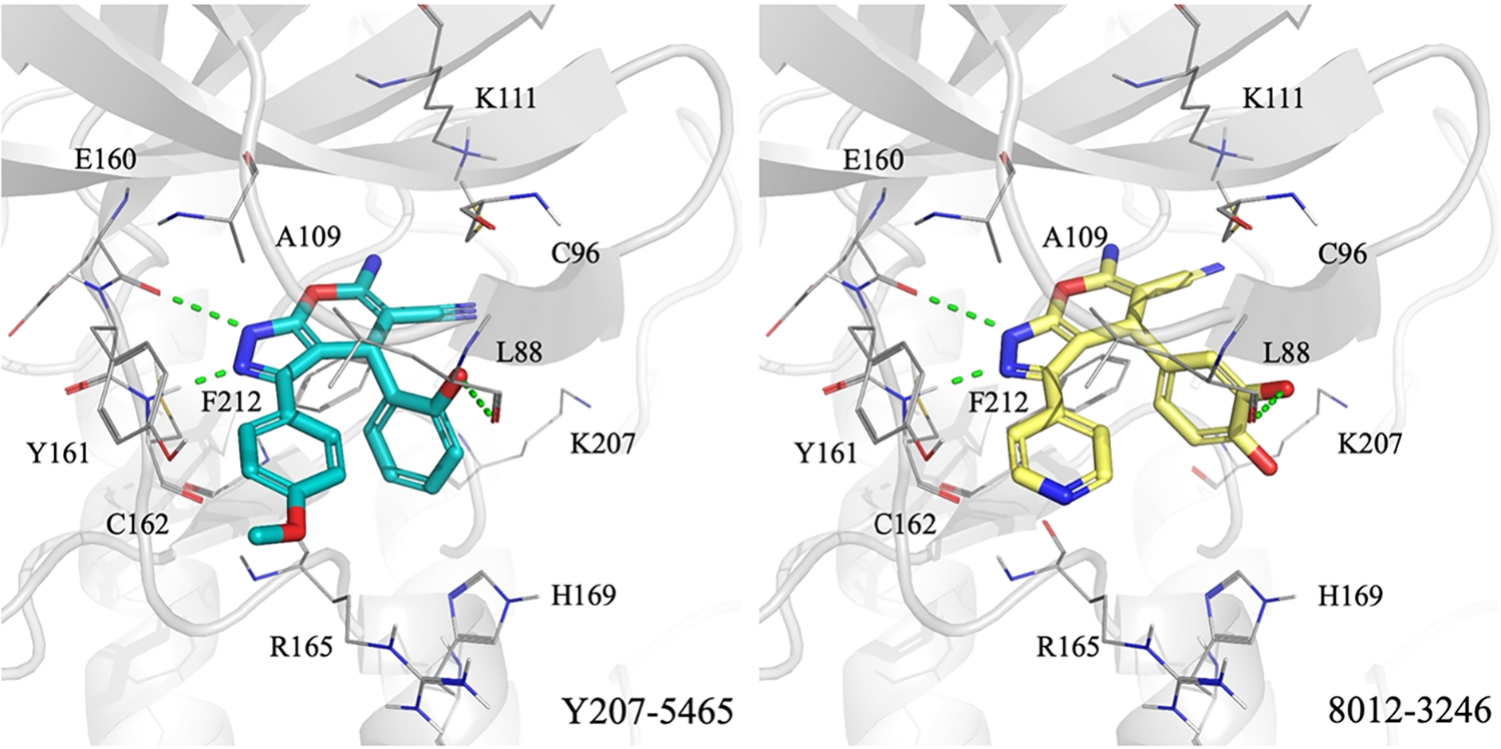
Docking poses of compounds Y207-5465 and 8012-3246 within the PLK2 binding site. Docked conformations of Y207-5465 (blue) and 8012-3246 (yellow) show favorable occupancy of the PLK2 binding site (gray). Hydrogen bonds are shown as green dashed lines. Binding site residues are labeled. The compound structures are segmented into three regions: Core scaffold (red), R_1_ substituent (yellow), and R_2_ substituent (blue).

### SAR Analysis

To better understand the SAR, we grouped the analogs into four categories based on their R_2_ substituents (Figure 4). The effects of R_1_ and R_2_ modifications on molecular interactions and inhibitory activity were analyzed for each group (Figure 4A). In Group A, which includes analogs with a 4-methoxyphenyl R_2_ group, Y207-5465 exhibited the highest activity (97%) due to favorable interactions at both the R_1_ and R_2_ sites described above. In comparison, K638-0003 retains the L88 hydrogen bond but loses hinge interactions with Y161 and C162, possibly due to altered orientation caused by its R_1_ substituent, resulting in reduced activity (24%) (Figure 4B,D). In K638-0019, the R_1_ substituent was modified from a hydroxyl group to a thiophene-2-carboxylate group. This larger functional group prevents the formation of a hydrogen bond with L88 and extends outward from the protein surface, thereby disrupting interactions with hinge residues Y161 and C162, resulting in reduced activity (13%). K638-0024, which contains bulky R_1_ groups, fails to maintain the L88 hydrogen bond and also loses hydrophobic contacts with Y161 and C162, likely due to steric hindrance, leading to further reductions in activity (11%) (Figure 4B,D). These findings highlight the importance of both the L88 hydrogen bond and hydrophobic interactions with Y161 and C162 for high potency (Figure 4C). Compounds with a phenolic moiety at the R_1_ site exhibit higher inhibitory activity than those with a bulkier substituent (Figure 4D).

**Figure 4:**
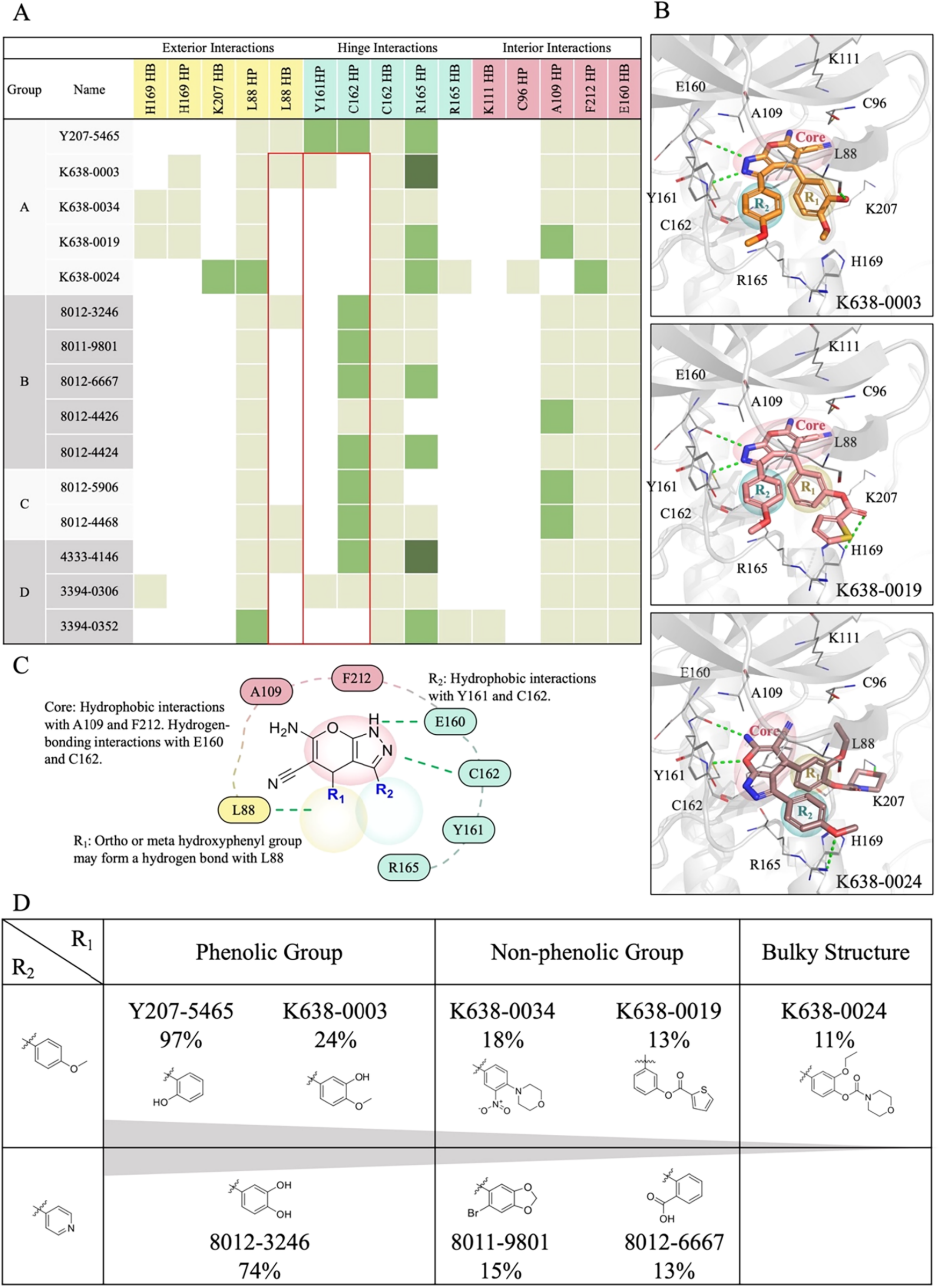
Structure–activity relationship analysis of analogs. (A) Interaction profiles of the analogs categorized into four groups based on their R_2_ substituent structures. Residue interactions with core, R_1_, and R_2_ are classified. HB and HP denote hydrogen-bonding and hydrophobic interactions, respectively. The number of interactions formed by each compound is represented by a color scale: light green (one), green (two), and dark green (three). (B) Docked conformations of the analogs showing the key interactions within the PLK2 binding site. Hydrogen bonds are shown as green dashed lines. Binding site residues are labeled. The compound structures are segmented into three regions: Core scaffold (red), R_1_ substituent (yellow), and R_2_ substituent (blue). (C) Interaction diagram in the PLK2 binding site. Hydrogen bonds are shown as green dashed lines. Residues from the exterior, interior, and hinge regions are colored yellow, red, and blue, respectively. (D) Key moieties affecting activity in the SAR analysis are summarized in the table.

In Group B, which contains analogs with a pyridyl R_2_ group, 8012-3246 showed 74% inhibition due to the presence of a 3,4-dihydroxyphenyl R_1_ group that forms a hydrogen bond with L88 and hydrophobic interactions between its R_2_ moiety and residue C162. In contrast, other analogs in this group, such as 8011-9801 and 8012-6667, lack these key hinge or exterior interactions and exhibited much lower activity (15% and 13%, respectively). Groups C and D include compounds with R_1_ and R_2_ substituents that fail to establish the key interactions. For example, 8012-5906 and 3394-0352 lose both the hydrogen bond with L88 and the hydrophobic interactions with Y161 and C162, resulting in reduced activity. These results suggest that potent PLK2 inhibition requires a hydrogen bond at L88, mediated by an ortho- or meta-hydroxyphenyl R_1_ group, as well as hydrophobic interactions between the R_2_ scaffold (e.g., methoxyphenyl or pyridyl) and residues Y161 and C162 (Figure 4C).

### Structural Diversity of PLK2 Inhibitors

To evaluate the structural novelty of the compounds Y207-5465 and 8012-3246, a similarity matrix was generated using 30 structurally diverse PLK2 inhibitors. The matrix contained Tanimoto scores for all molecule pairs (Figure S2). As analogs, Y207-5465 and 8012-3246 exhibited the highest mutual similarity, with a Tanimoto score of 0.523. In contrast, comparison of the compounds with known PLK2 inhibitors yielded no significant structural similarity or clustering patterns. Visual inspection revealed the top-scoring structural matches: CHEMBL3975634 (Tanimoto score of 0.137 with Y207-5465) and CHEMBL2205426 (Tanimoto score of 0.123 with 8012-3246). This analysis suggests that Y207-5465 and 8012-3246 are structurally distinct from previously reported PLK2 inhibitors.

### PLK2 Inhibitor Exhibits Potent Anticancer Activity in CRC Cells

To assess the anticancer efficacy of PLK2 inhibitors, we conducted cell viability assays using CRC cell lines HT-29 and HCT-116. Among the two compounds tested, 8012-3246 demonstrated potent cytotoxicity, while Y207-5465 showed only limited activity (Figure 5A). The treatment with 8012-3246 at concentrations of 10 and 30 μM resulted in significant reductions in cell viability in both cell lines, with calculated IC_50_ values of 7.97 μM for HT-29 and 17.67 μM for HCT-116, respectively (Figure 5A,B). Based on these results, 8012-3246 was selected for further evaluation. To further explore the anticancer mechanism of PLK2 inhibition, a BrdU incorporation assay was conducted to assess its effect on cancer cell proliferation. The results demonstrated that 8012-3246 markedly inhibited DNA synthesis in a dose-dependent manner, indicating an antiproliferative effect. The GI_50_ values for growth inhibition were determined to be 3.28 μM for HT-29 and 6.62 μM for HCT-116 (Figure 5C). Moreover, in colony formation assays, the treatment with 3 μM of 8012-3246 led to a visible reduction in colony numbers, while higher concentrations, 10 and 30 μM, resulted in a pronounced suppression of colony-forming ability in both cell lines (Figure 5D,E). These results demonstrated that 8012-3246 exhibits strong anticancer activity by inhibiting cell viability, proliferation, and clonogenic growth in CRC cells.

**Figure 5:**
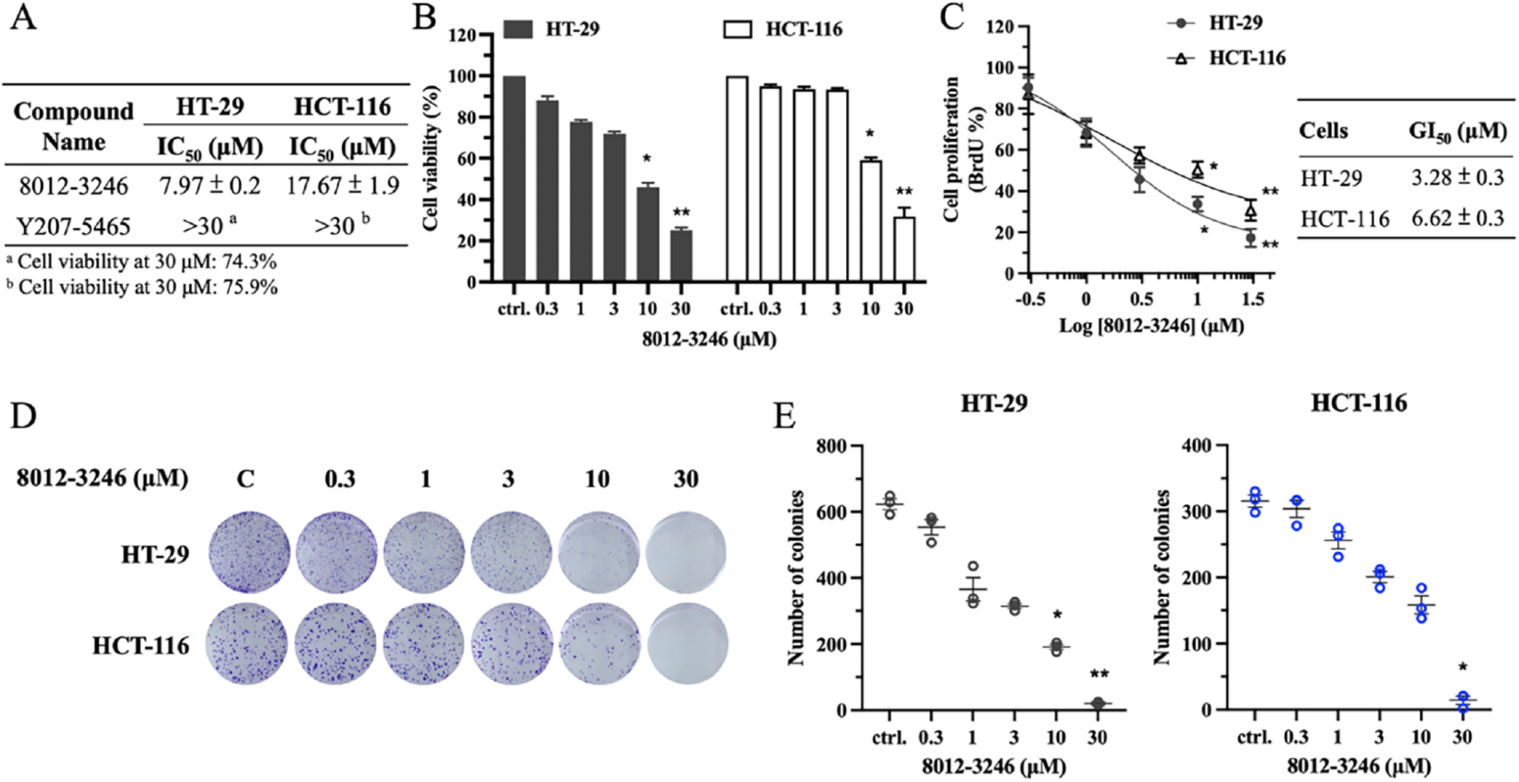
**T**he effect of PLK2 inhibitors in CRC cells. HT-29 and HCT-116 cells were treated with compounds at 0.3, 1, 3, 10, and 30 μM for 72 h. (A) The IC_50_ values of cell viability were calculated based on MTT assay results.^a,b^ The percentage of cell viability following Y207-5465 treatment at 30 μM is also shown. (B) Cell viability profile under 8012-3246 treatment. (C) Cell proliferation response to 8012-3246. DNA synthesis activity was measured by BrdU assay following compound treatment for 72 h. The calculated GI_50_ values of cell proliferation are presented. (D) Colony formation analysis. Cancer cells were treated with compounds at 0.3, 1, 3, 10, and 30 μM for 12 days. (E) The quantitative results of colony numbers. *p < 0.05, **p < 0.001 compared to the control (ctrl., untreated) group.

### 8012-3246 Inhibits PLK2 Kinase Activity and Downstream Signaling in Cancer Cells

The enzyme-based assay confirmed that 8012-3246 exhibited inhibitory activity against PLK2. To further validate the mechanism of action within a cellular context, we examined the effect on PLK2-mediated signaling in CRC cells. Given the specificity of 8012-3246 for PLK2, we analyzed the phosphorylation status of GSK3β, a well-established downstream substrate of PLK2. Western blot analysis revealed that 8012-3246 led to a significant and dose-dependent reduction in GSK3β phosphorylation at Ser9 (Figure 6). This result confirmed that 8012-3246 effectively inhibited PLK2 kinase activity and the downstream signaling in cancer cells. Collectively, these findings support that 8012-3246 suppresses PLK2 signaling and CRC cell growth, thus providing a promising therapeutic strategy for halting cancer progression.

**Figure 6:**
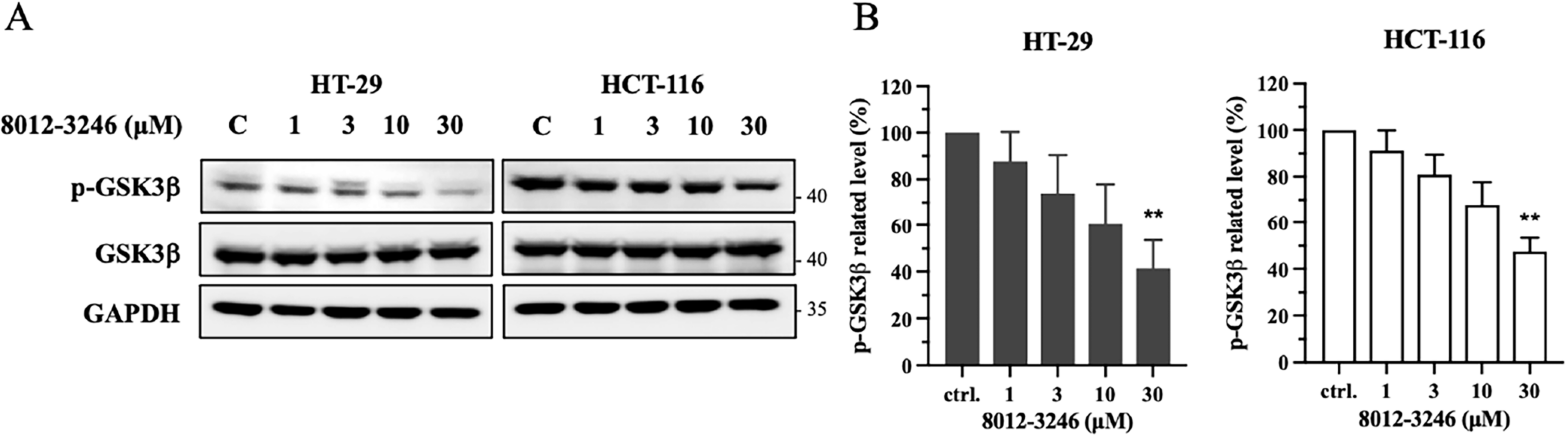
The effect of PLK2 inhibitors in CRC cells. HT-29 and HCT-116 cells were treated with 8012-3246 at 1, 3, 10, and 30 μM for 2 h. (A) Downstream signaling expression. The phosphorylation level of GSK3β was assessed by Western blotting. (B) The corresponding quantitation of GSK3β phosphorylation. *p < 0.05, **p < 0.001 compared to the control (ctrl., untreated) group.

### Selectivity Profile of Compound 8012-3246

The development of kinase inhibitors is often hindered by challenges in achieving selectivity, as off-target inhibition can lead to unexpected effects. To evaluate the selectivity of 8012-3246, the compound was tested against a panel of 40 kinases representing diverse families within the human kinome. At a concentration of 10 μM, 8012-3246 showed minimal inhibition across the panel. Only a few kinases, including MARK2 (26%), STK3 (26%), BRAF (23%), and WEE1 (21%), exhibited modest inhibition, and none exceeded 50% (Table 4). In contrast to many PLK2 inhibitors that exhibit broad activity across multiple targets, 8012-3246 demonstrated a more selective inhibition profile. For example, CHEMBL5181620 inhibits not only PLK2 but also DAPK3 and DYRK1A, with IC_50_ values of 125, 578, and 917 nM, respectively.^34^ Similarly, BI-2536, which has completed a phase 2 clinical trial, has an IC_50_ value of 190 nM for mutated ALK.^35–37^ These findings suggest that PLK2 is the primary target of 8012-3246 and support its potential use as a selective probe for investigating PLK2-associated signaling pathways. Moreover, 8012-3246 represents a promising starting point for further development as a therapeutic agent.

**Table 4:** Selectivity Profile of Compound 8012-3246

kinase	% inhibition at 10 μM	kinase	% inhibition at 10 μM
MARK2	26%	PTK2	6%
STK3	26%	RPS6KA3	6%
BRAF	23%	TYK2	6%
WEE1	21%	DAPK1	4%
CAMKK1	20%	ICK	4%
DYRK3	19%	MAP2K1	4%
BTK	14%	CDK6	0%
FGFR2	13%	EPHA3	0%
CAMK1G	12%	IRAK4	0%
CDK1	11%	LCK	0%
STK24	10%	MAPK13	0%
CLK3	9%	MAPK8	0%
CSNK1D	9%	MAPKAPK2	0%
PLK4	9%	MINK1	0%
CDK7	8%	MKNK1	0%
ROCK1	8%	PHKG2	0%
DCLK1	7%	PLK1	0%
FLT3	6%	PLK3	0%
PAK2	6%	SRC	0%
PRKACA	6%	VRK2	0%

## Discussion

PLK2 has emerged as a promising therapeutic target in CRC. In this study, we developed a drug design framework that integrates computational modeling with experimental validation to identify novel PLK2 inhibitors. Pharmacological interactions from multiple PLK2 structures were first identified using known inhibitors, and a consensus model was constructed to enhance hit rates during screening. Previous studies have demonstrated that incorporating multiple protein structures can improve hit rates in virtual screening. For instance, ensemble docking against aminergic G protein-coupled receptors achieved an eight-to 17-fold enrichment,^38,39^ with similar improvements observed for the SARS-CoV-2 receptor-binding domain.^40,41^ Similarly, our consensus model increased the AUC from 0.906 to 0.930 and led to the identification of two novel PLK2 inhibitors. Notably, the top compound, Y207–5465, improved from modest ranks in individual models to 16th in the consensus ranking. This compound was experimentally validated as a submicromolar PLK2 inhibitor with high kinase selectivity and antiproliferative activity in CRC cell lines. These findings support the framework of incorporating pharmacological interaction analysis across multiple structures to enhance the hit rate in virtual screening.

In this study, we observed a marked difference in sensitivity to the PLK2 inhibitor 8012-3246 between two CRC cell lines, HT-29 and HCT-116. HT-29 cells exhibited significantly greater susceptibility, as indicated by lower IC_50_ and GI_50_ values in both viability and proliferation assays (Figure 5). This differential response may be attributed to intrinsic genetic and molecular differences between the two cell lines. Notably, HT-29 carries a truncating mutation in the APC gene, resulting in constitutive activation of the Wnt/β-catenin signaling pathway, while HCT-116 retains wild-type APC.^42,43^ Since PLK2 regulates downstream effectors such as GSK3β, which is involved in β-catenin degradation, inhibition of PLK2 in APC mutant cells like HT-29 may further disrupt Wnt signaling balance and enhance oncogenic stress.^4,44^ Additionally, HT-29 cells harbor mutant p53, potentially impairing their ability to initiate cell cycle arrest and DNA repair.^45^ This may increase reliance on PLK2-mediated survival pathways.^46^ In contrast, HCT-116 cells express functional p53 and may compensate for PLK2 inhibition through robust checkpoint control and alternative adaptive mechanisms. These findings suggest that APC and p53 status could serve as predictive indicators of cellular response to PLK2 targeted therapies, highlighting the importance of genetic background in therapeutic sensitivity.

Pharmacological interactions often involve residues with key roles in inhibitor potency, biological function, or evolutionary conservation. Pharmacological interactions identified in this study include a hydrogen bond with residue C162 and hydrophobic interactions with residues L88, A109, R165, and F212. A sequence conservation analysis of PLK2 was conducted via the ConSurf database,^47,48^ which assigns each residue a conservation score ranging from variable (1) to highly conserved (9). The analysis revealed that A109, C162, and F212 are among the most highly conserved residues with scores of 9, while L88 and R165 are also conserved with scores of 8, highlighting their essential role in maintaining PLK2 kinase activity (Figure S3). Previous studies have demonstrated that these residues play an important role in inhibitor binding. For instance, highly conserved residues C162 and F212 stabilize the inhibitor scaffold within the PLK2 binding site.^16^ In addition, residues L88 and R165 anchor inhibitors by sandwiching their aromatic rings through hydrophobic interactions.^49^ The SAR analysis in this study also confirmed that interacting with highly conserved L88 and C162 may improve the potency (Figure 4). In summary, potent inhibitors constantly engage highly conserved residues, which are crucial for inhibitor binding.

The safety profiles of compounds Y207-5465 and 8012-3246 were evaluated using ProTox 3.0,^50^ a computational tool for in silico toxicity prediction. Both compounds were assigned to toxicity class IV by ProTox 3.0, indicating low acute toxicity in animal models. They also exhibited low predicted risks across most toxicological end points (Figure S4), including neurotoxicity, immunotoxicity, and mutagenicity. Although several end points were predicted as active, the associated probabilities remained low and did not exceed the commonly accepted concern threshold of 0.7, suggesting a relatively low likelihood of toxicity. Taken together, these predictions suggest that Y207-5465 and 8012-3246 demonstrate favorable predicted safety profiles and are unlikely to present significant toxicity risks. However, the predictions need to be validated through in vitro and in vivo toxicological studies to confirm their suitability for therapeutic development.

## Conclusion

High PLK2 expression in CRC is strongly associated with chemoresistance, rapid disease progression, and reduced survival, making PLK2 inhibition a promising strategy to improve prognosis. In this study, we employed a structure-based approach to construct a consensus model integrating pharmacological interactions from multiple PLK2 crystal structures, thereby enhancing virtual screening accuracy. This model identified two inhibitors, among which 8012-3246 demonstrated potent PLK2 inhibition, marked cytotoxicity, and strong antiproliferative effects in CRC cells. In addition, the compound exhibited high selectivity. Mechanistic analysis revealed that 8012-3246 suppressed GSK3β phosphorylation, effectively inhibiting downstream PLK2 signaling. These findings validate the utility of our consensus modeling strategy for kinase inhibitor discovery and support PLK2 inhibition as a viable therapeutic approach for CRC.

## Supplementary Material



## Data Availability

The cocrystal structure used for virtual screening was downloaded from the Protein Data Bank,^22^ while the compound library was downloaded from ChemDiv (https://www.chemdiv.com). Supplementary data related to this study are accessible via the GitHub repository (https://github.com/CharlieYang-gif/PLK2-inhibitors.git). Molecular docking was performed using Glide in Maestro,^21^ and the docking poses of compounds were analyzed and visualized with PyMol. Docking poses of the protein and compounds are provided in the repository as.pdb and.sdf files, respectively.

## Notes

Performance tables for PLK2 structural models and consensus predictions; dose–response kinase inhibition curves; matrix of structural similarities among hit compounds; residue conservation profiles for PLK2 residues (PDF)
