# Supplementary material for: Consensus Pharmacological Interactions for PLK2 Inhibitor Identification in Colorectal Cancer Treatment
Source: J Chem Inf Model. 2025 Dec 17;66(1):577–90. doi: 10.1021/acs.jcim.5c02197 (PMC12801292; doi:10.1021/acs.jcim.5c02197)
Supplement: Supplementary file 1 [file ci5c02197_si_001.pdf]

Consensus Pharmacological Interactions for PLK2 Inhibitor Identification in Colorectal Cancer Treatment

Yi-Wen Wu[1,#], Chun-Lin Yang[1,#], Tony Eight Lin[1,2], Yun-Hsuan Yeh[1], Yu-Ting Fang-Chin[1,2], Tzu-Ying Sung[1], Shih-Chung Yen[3], Jui-Hua Hsieh[4], Cheng-Chih Chung[5,6], Shiow-Lin Pan[1,2,7,*], Kai-Cheng Hsu[1,2,7,8,*]

[1]Graduate Institute of Cancer Biology and Drug Discovery, College of Medical Science and Technology, Taipei Medical University, Taipei, 11031, Taiwan
[2]Ph.D. Program for Cancer Molecular Biology and Drug Discovery, College of Medical Science and Technology, Taipei Medical University, Taipei, 11031, Taiwan
[3]Warshel Institute for Computational Biology, School of Medicine, The Chinese University of Hong Kong (Shenzhen), Shenzhen, Guangdong, 518172, China
[4]Division of Translational Toxicology, National Institute of Environmental Health Sciences, National Institutes of Health, Durham, NC, 27709, USA
[5]Division of Cardiology, Department of Internal Medicine, School of Medicine, College of Medicine, Taipei Medical University, Taipei, 11031, Taiwan
[6]Division of Cardiovascular Medicine, Department of Internal Medicine, Wan Fang Hospital, Taipei Medical University, Taipei, 11696, Taiwan
[7]TMU Research Center of Cancer Translational Medicine, Taipei Medical University, Taipei, 11031, Taiwan
[8]Cancer Center, Wan Fang Hospital, Taipei Medical University, Taipei, 11031, Taiwan

[#]These authors contributed equally to this work.

*Corresponding authors

E-mail: piki@tmu.edu.tw (K.C. Hsu)

E-mail: slpan@tmu.edu.tw (S.L. Pan)

Table S1. Performance of five PLK2 structures.

PDB	RMSD (Å)	Docking Score ROC AUC	Pharmacological Score ROC AUC
4I5M	1.01	0.796	0.908
4I5P	0.94	0.842	0.918
4I6B	0.99	0.800	0.847
4I6F	0.75	0.841	0.906
4I6H	1.12	0.863	0.896

Table S2. Performance of consensus models based on combinations of PLK2 structures.

PDB	ROC AUC
4I5M + 4I5P	0.925
4I5M + 4I6F	0.920
4I5P + 4I6F	0.925
4I5M + 4I5P + 4I6F	0.930

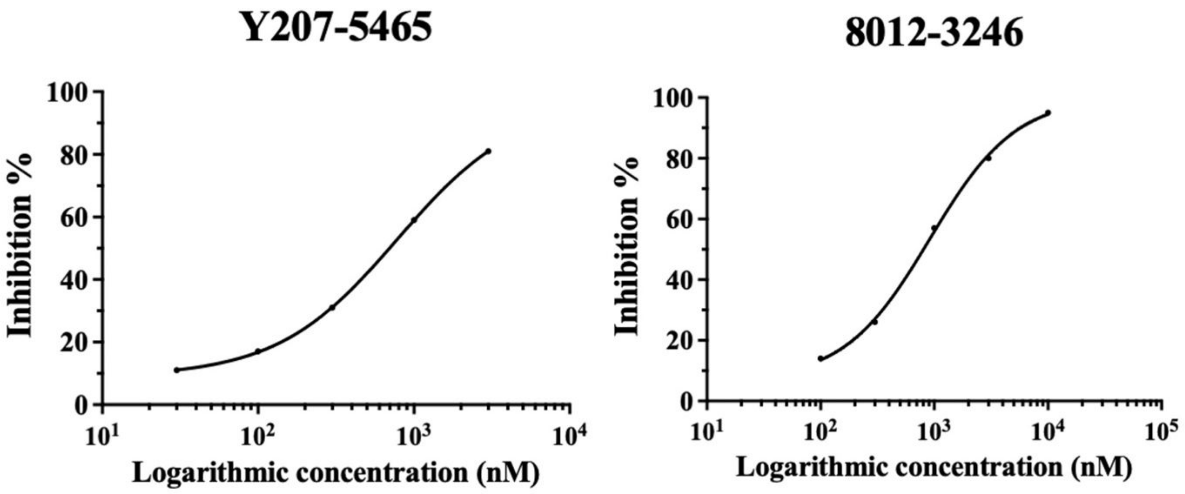

Figure S1. Dose-Dependent Inhibition of Kinase Activity.

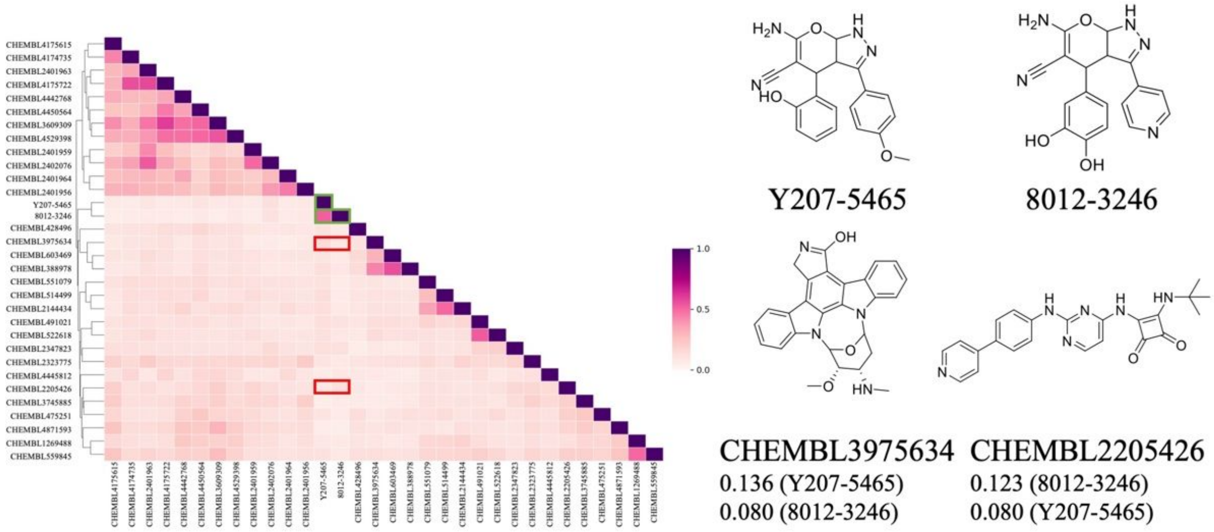

Figure S2. Similarity scores of known PLK2 inhibitors. A similarity matrix was generated between the hit molecules and 30 known and structurally diverse PLK2 inhibitors. Molecules with the highest Tanimoto scores remain the two hit molecules. Known PLK2 inhibitors did not produce a score greater than 0.4 when paired to the hit molecules. The green and red box highlights the matrix position of the hit molecules and the next highest scoring PLK2 inhibitor pair.

A

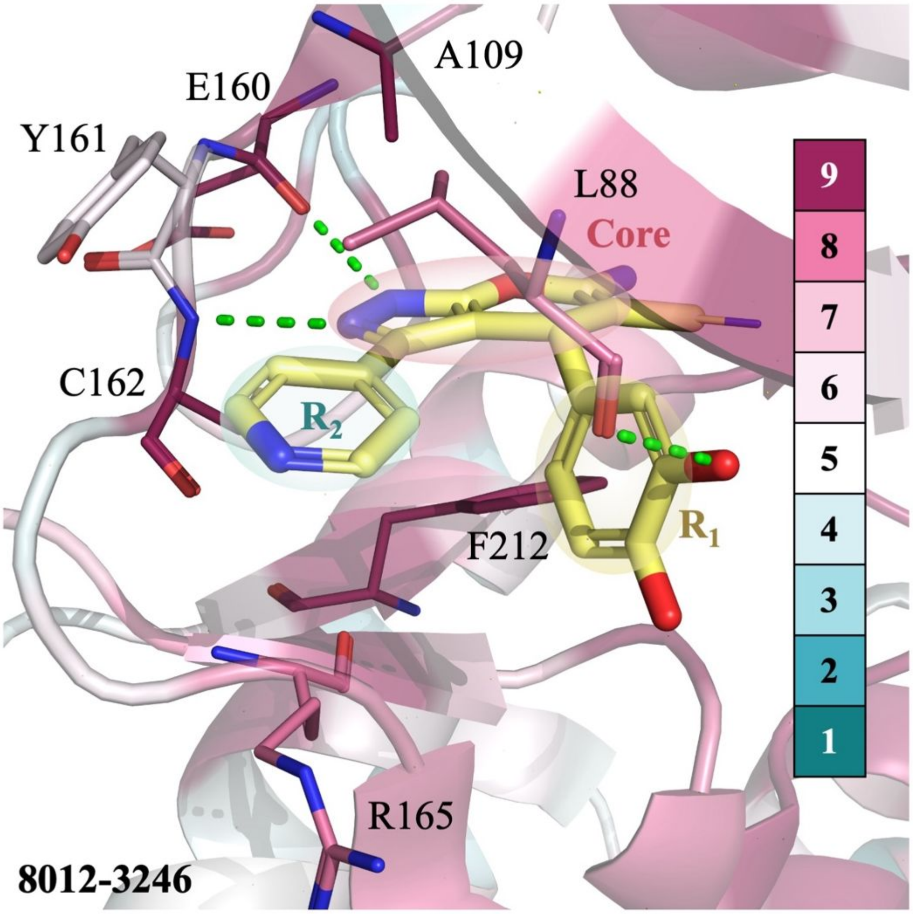

B

Protein-ligand Interactions	A109 M	C162 HB	F212 P	C162 M	E160 HB	L88 M	R165 M	L88 HB
Interaction Frequency	100%	83%	80%	70%	23%	93%	93%	57%
Conservation Grades	9	9	9	9	9	8	8	8
8012-3246 Interactions	1	1	1	2	1	1	1	1

Figure S3. Conservation of residue positions in PLK2. (A) Representation of PLK2 (PDB ID: 4I6F) colored by evolutionary conservation, with maroon denoting the most highly conserved residues (score 9) and cyan denoting the most variable (score 1). The docking pose of 8012-3246 is shown for reference. The hydrogen bonds are shown as green dashed lines. (B) Summary table of protein-ligand interactions, including frequencies, conservation scores, and the interactions observed for 8012-3246. P, M, and HB stand for pi-stacking interaction, mixed hydrophobic interaction, and hydrogen bonding interaction, respectively.

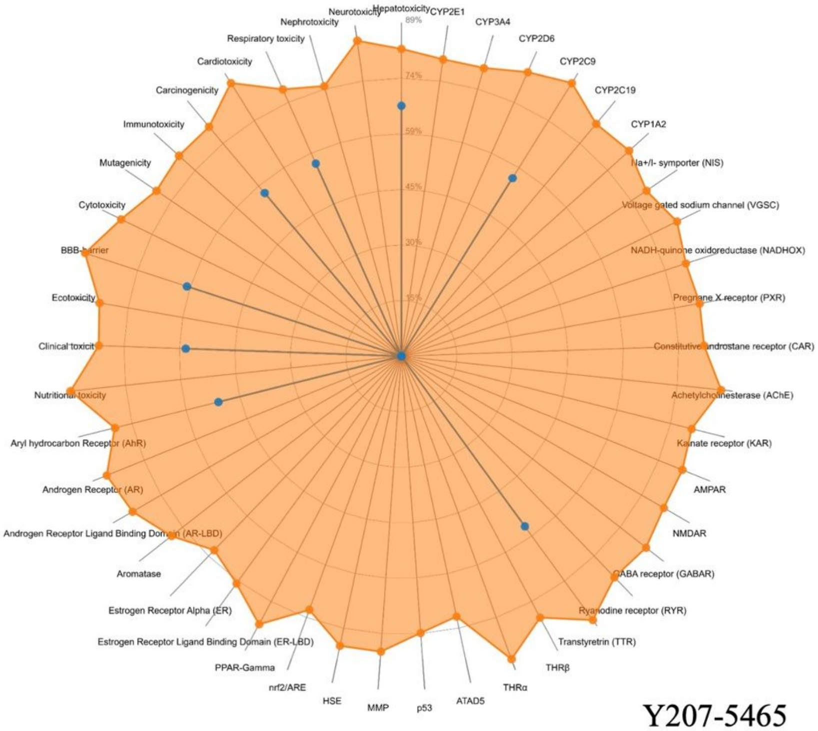

Y207-5465

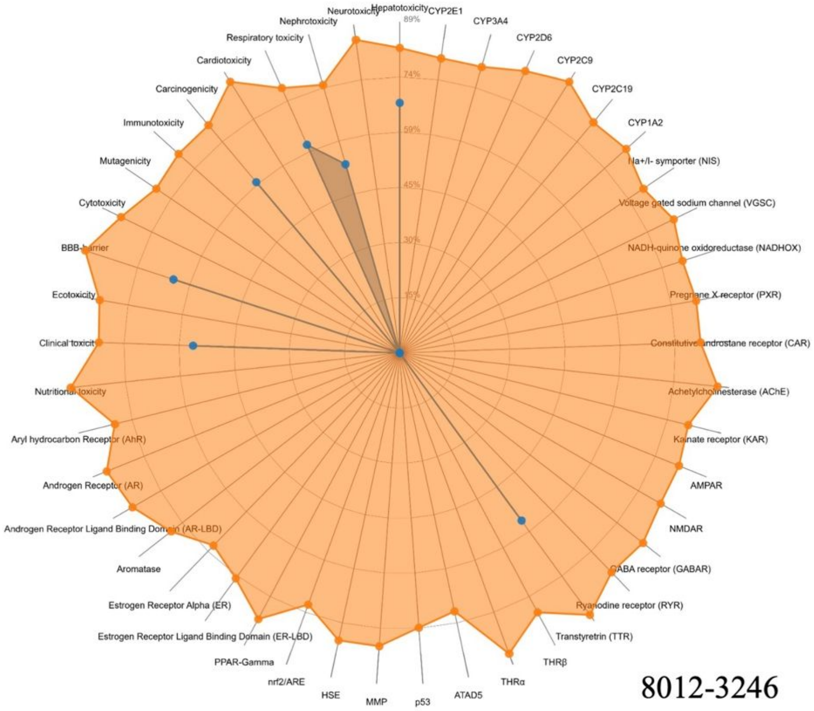

8012-3246

Figure S4. Toxicity radar chart. Orange lines and dots indicate the average prediction confidence for the active class. For the input compound, only endpoints predicted as active are displayed in blue lines and dots, representing their predicted probabilities. Endpoints without blue markers are predicted as inactive.
